# New Advertisements

**Published:** 1894-01

**Authors:** 


### NEW ADVERTISERS.

We desire to call our readers special attention to the following New Advertisements in this issue. We will have something more to say about these advertisements in the future. We can most heartily recommend them.

McArthur Hypophosphite Co.; Scott & Browne; Walker-Green Pharmaceutical Co.; Physicians Mutual Manf. Co.; D. L. Dowd's; H. H. Ballard; W. Scott Marshall; Stevenson & Jester.

---

### TO SUBSCRIBERS.

We again call our subscribers attention to the fact that a good many of them have failed to remit for last years subscription. Please do so at once.

